# Exogenous Hydrogen Sulfide (H_2_S) Protects Alveolar Growth in Experimental O_2_-Induced Neonatal Lung Injury

**DOI:** 10.1371/journal.pone.0090965

**Published:** 2014-03-06

**Authors:** Arul Vadivel, Rajesh S. Alphonse, Lavinia Ionescu, Desiree S. Machado, Megan O’Reilly, Farah Eaton, Al Haromy, Evangelos D. Michelakis, Bernard Thébaud

**Affiliations:** 1 Ottawa Hospital Research Institute, Sprott Center for Stem Cell Research, Regenerative Medicine Program and Children’s Hospital of Eastern Ontario, University of Ottawa, Ottawa, Ontario, Canada; 2 Department of Pediatrics, School of Human Development, Women and Children’s Health Research Institute, Cardiovascular Research Center and Pulmonary Research Group, University of Alberta, Edmonton, Canada; University of Giessen Lung Center, Germany

## Abstract

**Background:**

Bronchopulmonary dysplasia (BPD), the chronic lung disease of prematurity, remains a major health problem. BPD is characterized by impaired alveolar development and complicated by pulmonary hypertension (PHT). Currently there is no specific treatment for BPD. Hydrogen sulfide (H_2_S), carbon monoxide and nitric oxide (NO), belong to a class of endogenously synthesized gaseous molecules referred to as gasotransmitters. While inhaled NO is already used for the treatment of neonatal PHT and currently tested for the prevention of BPD, H_2_S has until recently been regarded exclusively as a toxic gas. Recent evidence suggests that endogenous H_2_S exerts beneficial biological effects, including cytoprotection and vasodilatation. We hypothesized that H_2_S preserves normal alveolar development and prevents PHT in experimental BPD.

**Methods:**

We took advantage of a recently described slow-releasing H_2_S donor, GYY4137 (morpholin-4-ium-4-methoxyphenyl(morpholino) phosphinodithioate) to study its lung protective potential *in vitro* and *in vivo*.

**Results:**

*In vitro*, GYY4137 promoted capillary-like network formation, viability and reduced reactive oxygen species in hyperoxia-exposed human pulmonary artery endothelial cells. GYY4137 also protected mitochondrial function in alveolar epithelial cells. *In vivo*, GYY4137 preserved and restored normal alveolar growth in rat pups exposed from birth for 2 weeks to hyperoxia. GYY4137 also attenuated PHT as determined by improved pulmonary arterial acceleration time on echo-Doppler, pulmonary artery remodeling and right ventricular hypertrophy. GYY4137 also prevented pulmonary artery smooth muscle cell proliferation.

**Conclusions:**

H_2_S protects from impaired alveolar growth and PHT in experimental O_2_-induced lung injury. H_2_S warrants further investigation as a new therapeutic target for alveolar damage and PHT.

## Introduction

Preterm birth, respiratory distress syndrome (RDS), and bronchopulmonary dysplasia (BPD), the chronic lung disease of prematurity, continue to be important causes of morbidity and mortality in the neonatal intensive care unit [1]. Despite improvements in perinatal care, the incidence of BPD remains unchanged [2]. Preterm birth before 28 weeks of gestation interrupts the normal sequence of lung growth leading to impaired alveolar and lung vascular development [3]. Emerging evidence suggests that BPD may have long-term respiratory complications that reach beyond childhood. Follow-up studies indicate that children and young adults who were born very preterm are at an increased risk of respiratory symptoms, poor lung function, lower exercise capacity [4] and pulmonary hypertension (PHT) [5], [6]. Currently, there is no effective treatment for BPD.

Hydrogen sulfide (H_2_S) has long been considered a noxious and toxic gas. Newly acquired evidence indicates potential biomedical applications for H_2_S. H_2_S is now recognized - along with carbon monoxide (CO) and nitric oxide (NO) - as an endogenous gaseous mediator exerting important physiological actions [7]. The role of H_2_S in the developing lung is unknown. The discovery that H_2_S is an endogenously produced gaseous second messenger capable of modulating many physiological processes including vasodilation and cytoprotection [7], much like NO, prompted us to investigate the potential of H_2_S as a lung-protective agent. Thus, we hypothesized that H_2_S would preserve alveolar development and prevents PHT in experimental oxygen-induced lung injury in newborn rats.

## Materials and Methods

All procedures were approved by the University of Alberta Animal Care and Use Committee.

### Human Pulmonary Artery Endothelial Cells (HPAECs) Network Formation Assay

The formation of cord-like structures by human pulmonary artery endothelial cells (HPAECs [ATCC, Manassas, VA]) was assessed in Matrigel-coated wells [8]. HPAECs (40,000 cells/well) were seeded into 48-well plates coated with Matrigel (BD Biosciences, Mississauga, ON) into groups of triplicates: (1) room air, (2) room air+GYY4137 (100 microM) (3) hyperoxia (95% O_2_), (4) hyperoxia+GYY4137 (100 microM) and incubated at 37°C for 8 h. GYY4137 (morpholin-4-ium-4-methoxyphenyl (morpholino) phosphinodithioate) is a recently described slow-releasing H_2_S donor (Cayman chemical, Ann Arbor, Michigan). Cord-like structures were observed using an inverted phase contrast microscope (Leica, Richmond Hill, ON, Canada) and quantified by measuring the number of intersections and the length of structures in random fields from each well using OpenLab (Quorum Technologies Inc, ON, Canada).

### HPAECs Viability Assay

After 48 hrs of culture in room air (control), hyperoxia (95% O_2_) or hyperoxia+100 microM GYY4137, HPAECs viability was evaluated by measuring the mitochondrial-dependent reduction of colorless 3-(4,5-Dimethylthiazol-2-yl)- 2,5- diphenyltetrazolium bromide (MTT) (Invitrogen, Eugene, Oregon, USA) to blue colored formazan which was dissolved in dimethyl sulfoxide and the absorbance of each sample was spectrophotometrically measured at 550 nm with a Spectra Max 190 (Molecular Devices) microplate reader [9].

### Intracellular Reactive Oxygen Species (ROS) Measurement

After 48 hrs of culture in hyperoxia (95% O_2_) or hyperoxia+100 microM GYY4137, ROS activity was evaluated in HPAECs by the cell-permeable fluorogenic probe 2′, 7′-Dichlorodihydrofluorescin diacetate (DCFH-DA) using ROS assay kit obtained from Cell Biolabs, Inc (STA-342, San Diego, CA). In brief, DCFH-DA is diffused into cells and is deacetylated by cellular esterases to non-fluorescent 2′, 7′-Dichlorodihydrofluorescin (DCFH), which is rapidly oxidized to highly fluorescent 2′, 7′-Dichlorodihydrofluorescein (DCF) by ROS. The fluorescence intensity is proportional to the ROS levels within the cell cytosol.

### Pulmonary Artery Smooth Muscle Cells (PASMCs) Proliferation

PASMCs were freshly isolated from adult Sprague-Dawley rats following an established protocol [10] and maintained in DMEM supplemented with 10% FBS and 1% PSF (complete DMEM). For the MTT assay, cells were seeded into plastic 24-well cell culture plates at a density of 20,000 cells/well. When PASMCs were ∼80% confluent, media was replaced with 500 µL complete DMEM containing 20 ng/mL of platelet-derived growth factor (PDGF) and/or 100 mM GYY4137. Media were changed daily. After 96 hours, media was aspirated and replaced with 500 µL of a 3-(4,5-Dimethylthiazol-2-yl)-2,5-diphenyltetrazolium bromide (MTT, 0.5 mg/mL). Cells were incubated for 2 hours at 37°C. MTT was removed and 10 µL of DMSO was placed on cells to dissolve the formazan crystals. A colorimetric plate reader was used to measure absorbance at 550 nm, which is directly correlated with the number of live cells in the sample [9].

### Oxygen-induced Lung Injury

Rat pups were exposed to room air (21%, control group) or hyperoxia (95% O_2_, BPD group) from birth to P14 in sealed Plexiglas chambers (BioSpherix, Redfield, NY) with continuous O_2_ monitoring [8]. Dams were switched every 48 hours between the normoxic and hyperoxic chambers to prevent damage to their lungs and provide equal nutrition to each litter. Litter size was adjusted to 12 pups to control for effects of litter size on nutrition and growth. Rat pups were sacrificed at P21 for prevention experiment and at P30 for rescue experiment with intraperitoneal pentobarbital, and lungs and heart were processed, according to the performed experiments.

### Experimental Protocol

Newborn rat pups were randomized to four groups: (1) room air; (2) room air+GYY4137; (3) hyperoxia (95% O2, BPD group); and (4) hyperoxia+GYY4137. GYY4137 (37.75 mg/kg/day [11]; Cayman chemical, Ann Arbor, Michigan diluted in sterile distilled water) was administered daily via intraperitoneal injection from P4 to P14 in the prevention study and from P14 to P24 in the rescue study.

### Lung Morphometry

Lungs were fixed with a 10% formaldehyde solution through the trachea at a constant pressure of 20 cm H_2_O. The trachea was then ligated and the lungs immersed in fixative overnight. Lungs were processed and embedded in paraffin. Serial step sections, 4 µm in thickness, were taken along the longitudinal axis of the lobe. The fixed distance between the sections was calculated so as to allow systematic sampling of 10 sections across the whole lobe. Lungs were stained with hematoxylin and eosin (H&E). Alveolar structures were quantified on a motorized microscope stage by using the mean linear intercept (MLI) as previously described [8], [12], [13].

### Echo-doppler

Pulmonary artery acceleration time, expressed as a ratio over the right ventricular ejection time (PAAT/RVET), was assessed by Doppler echocardiography as previously described [14].

### Right Ventricular Hypertrophy (RVH) and Pulmonary Artery Remodeling

Right ventricle and left ventricle plus septum were weighed separately to determine the right ventricle to left ventricle+septum ratio (RV/LV+S) as an index of RVH [12]. To assess pulmonary artery remodeling, the medial wall thickness (MWT) was calculated as (external diameter-lumen diameter)/vessel diameter using small vessels (30–100 µm) [12].

### Immunoblotting

Protein expression in whole lungs was measured with immunoblotting as previously described [8] using commercially available antibodies. The intensity of the bands was normalized to the intensity of a reporter protein (actin) using the Kodak Gel-doc system. Akt and phospho-Akt antibodies were obtained from Cell Signaling (Cat # 9272 and 9271, Danvers, MA), SIRT1 antibody from Santa Cruz Biotechnology Inc (sc-15404, Santa Cruz, CA), caspase 3 and CD31 from Abcam (ab13847 and ab24590, Cambridge, MA, USA).

### Immunohistochemistry

von Willebrand Factor (vWF) positive lung capillaries (30–100 µm) were quantified on a Zeiss (Imager.M2) microscope [15].

### Mitochondrial Function

Imaging was performed with a Zeiss LSM 510 confocal microscope (Carl Zeiss). Mitochondrial membrane potential (ΔΨm) was studied in live rat lung epithelial cells (RLE-6TN (ATCC® CRL-2300™ Manassas, VA, USA) as described [16] using tetramethyl-rhodamine methyl-ester perchlorate (TMRM) (10 nM/L, ×30 minutes, 37°C) from Molecular Probes, Eugene, OR. Mitochondrial superoxide production was measured using MitoSOX™ (5 µM, Molecular Probes, Eugene, OR). Fluorescein isothiocyanate (FITC) and tetramethylrhodamine isothiocyanate (TRITC)-conjugated (DAKO, Markham, Ontario) secondary antibodies were used in immunofluorescence. TMRM and MitoSOX™ intensity was measured in ten random fields per slide; a minimum of five slides per experiment was used.

### Statistical Analysis

Values are expressed as means (±SEM). Statistical comparisons were made with ANOVA. Post hoc analysis used a Fisher’s probable least significant difference test (Statview 5.1; Abacus Concepts, Berkeley, CA). A value of P less than 0.05 was considered statistically significant. All counting assessments were performed by investigators blinded to the experimental groups.

## Results

### H_2_S Preserves HPAECs Network Formation, Viability and Decreases ROS Generation in Hyperoxia

**Figure 1:**
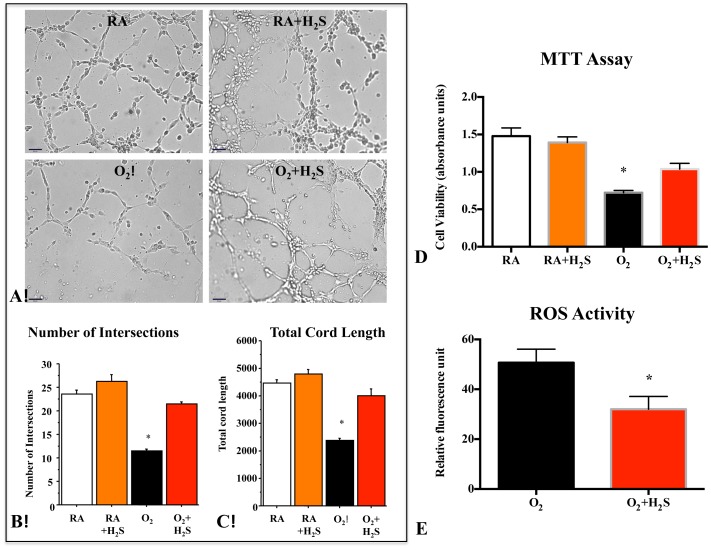
H_2_S protected human pulmonary artery endothelial cells (HPAECs) from O_2_-induced toxicity. (**A**) H_2_S promotes endothelial network formation. Quantitative assessment of cordlike structure formation shows a significant decrease in the number of intersects and the total length of cord-like structures in hyperoxia. H_2_S preserved the number of intersects (**B**) and total cord-structure length (**C**). (n = 3 per group, *P<0.0001 hyperoxia vs. other groups, scale bar 65 µm). (**D**) HPAECs were cultured for 48 hours in room air (Normoxia) or 95% hyperoxia. Mean data of cell viability as assessed by measuring the mitochondrial-dependent reduction of colorless 3-(4,5-dimethylthiazol-2-yl)-2,5-diphenyltetrazolium bromide (MTT) shows that hyperoxia significantly decreases HPAECs viability as compared with room air–exposed cells. H_2_S treatment significantly improved HPAECs viability in hyperoxia (n = 7, *P<0.001). (**E**) After 48 hours culture in hyperoxia (95%), ROS activity evaluated by measuring the dichlorofluorescein (DCF) shows that hyperoxia increases the ROS production in HPAECs, treatment with GYY4137 significantly decreased the ROS (n = 6/group, *P<0.005 Hyperoxia vs O_2_+H_2_S).

*In vitro,* HPAECs were exposed to room air or 95% O_2_ in serum-free Matrigel and assessed for the formation of cord-like networks (Figure 1A). Hyperoxia significantly decreased endothelial cord-like structure formation as assessed by the number of intersections (Figure 1B) and average tube length (Figure 1C). GYY4137 significantly counteracted the effect of O_2_ and promoted endothelial network formation (Figures 1B and C).

Viability of HPAECs was significantly decreased in hyperoxia significantly decreased compared to room air cultured HPAECs (Figure 1D). GYY4137 significantly improved HPAEC survival by ∼43% (P<0.001) in hyperoxia (Figure 1D).

As shown by dichlorofluorescein oxidation assay (Figure 1E), GYY4137 treatment of HPAECs prevented cellular ROS production in hyperoxia compared with untreated hyperoxic control.

These *in vitro* assays formed the rationale to further investigate the therapeutic potential of the H_2_S donor GYY4137 in an experimental model of hyperoxia-induced neonatal rat lung injury mimicking BPD.

### GYY4137 Treatment Preserves Alveolar Growth in O_2_-induced Lung Injury in Neonatal Rats

**Figure 2:**
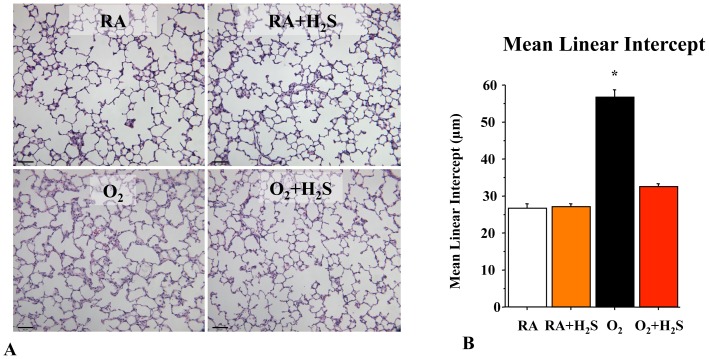
*In vivo* H_2_S treatment prevents arrested alveolar growth in experimental O_2_-induced lung injury. Representative (**A**) hematoxylin and eosin (H&E)-stained (scale bar 130 µm) lung sections at P21 showing larger and fewer alveoli in hyperoxia-exposed lungs as compared with lungs of room air housed rat pups. Treatment of hyperoxia-exposed animals with H_2_S preserved alveolar structure. (**B**) The mean linear intercept confirms arrested alveolar growth in untreated O_2_-exposed animals and preserved alveolar structure with H_2_S treatment (n = 5 per group, *P<0.0001 hyperoxia vs. other groups).

To test the therapeutic potential of GYY4137 *in vivo*, neonatal rats exposed for 14 days to hyperoxia were treated with daily intraperitoneal injections of GYY4137 from day P4 to P14. Hyperoxia induced a histological pattern reminiscent of human BPD, characterized by airspace enlargement with simplified and fewer alveolar structures as shown by representative hematoxylin and eosin (H&E) stained sections (Figure 2A). Treament with GYY4137 from P4–P14 preserved alveolar formation as quantified by the mean linear intercept (Figure 2B). GYY4137 treatment had no adverse effects on lung architecture in control animals.

### GYY4137 Treatment Preserves Lung Vascular Growth in O_2_-induced Lung Injury

**Figure 3:**
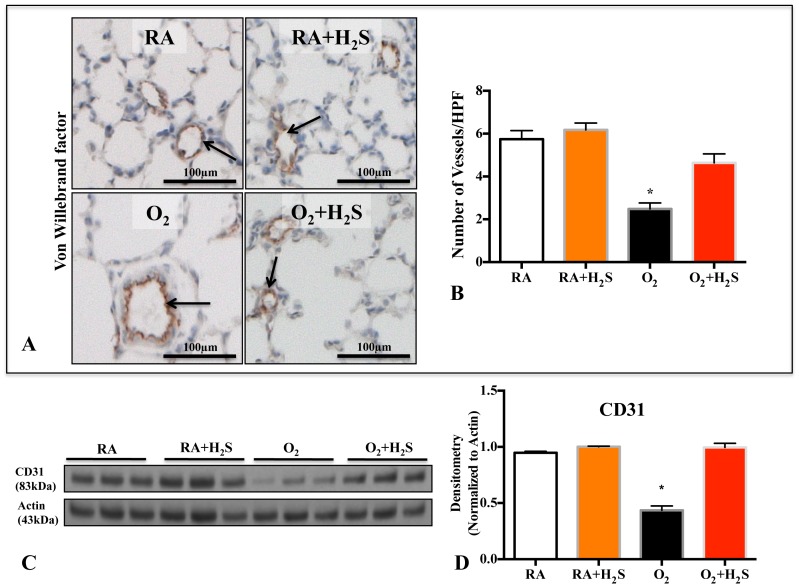
*In vivo* H_2_S treatment prevents O_2_-induced arrested lung vascular growth. A. Representative photomicrographs showing von Willebrand (vWF) factor staining (brown) in RA (room air), RA+H_2_S, hyperoxia (O_2_) and O_2_+H_2_S exposed lungs. Arrows highlight vWF-positive vessels; scale bars represent 100 µm. B. Mean data quantifying the number of vWF positive vessels between groups. The decrease in the number of vessels per high-power field (HPF) after hyperoxia exposure was prevented by H_2_S treatment (n = 5–7/group, *P<0.005 Hyperoxia vs O_2_+H_2_S). C. Representative immunoblot and densitometric (D) analysis for endothelial marker CD31 in lung homogenates from control and H_2_S treated animals. H_2_S treatment preserved the expression of CD31 in hyperoxic rats compared with hyperoxic control (n = 3/group, *P<0.005 Hyperoxia vs O_2_+H_2_S).

Hyperoxia also lead to an arrest in lung vascular growth as demonstrated by decreased vWF positive lung vessels (Figure 3A, B) and CD31 lung protein expression (Figure 3C, D). H_2_S treatment attenuated the loss of vWF positive cells (Figure 3A, B) and CD31 expression (Figure 3C–D).

### GYY4137 Reduces PHT Associated with O_2_-induced Lung Injury

**Figure 4:**
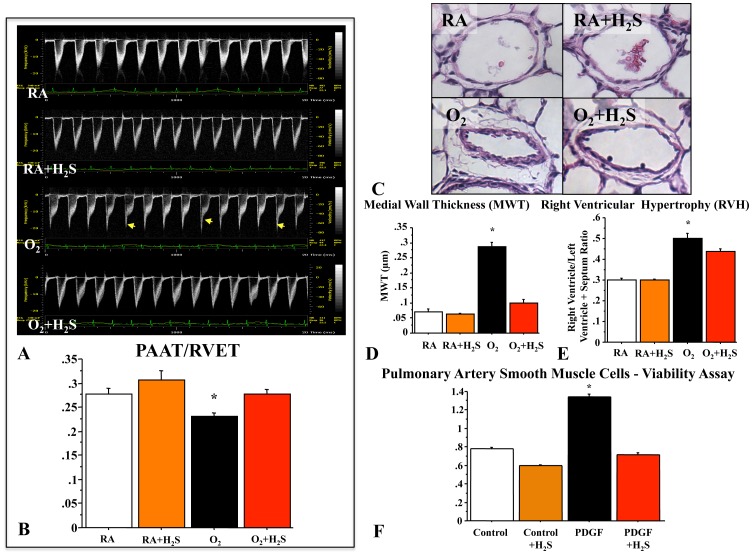
H_2_S prevents pulmonary hypertension associated with O_2_-induced lung injury. (**A**) Pulmonary arterial acceleration time/right ventricular ejection time (PAAT/RVET). Representative echo Doppler and mean PAAT/RVET showing a characteristic notch indicating PHT (**arrow**) in hyperoxic-exposed rat pups and a significantly decreased PAAT/RVET as compared with rat pups housed in room air. (**B**) H_2_S significantly increased PAAT/RVET as compared with untreated hyperoxic rat pups (n = 6 animals per group, *P<0.005 hyperoxia vs. other groups). (**C**) Pulmonary arterial medial wall thickness (MWT). Representative H&E stained sections of pulmonary arteries from the four experimental groups and % mean MWT. Hyperoxic-exposed rats had a significant increase in %MWT as compared with room air–housed rat pups. (**D**) H_2_S significantly reduced %MWT (n = 5 animals per group, *P<0.0001, hyperoxia vs. other groups, scale bar 65 µm). (**E**) Right ventricular hypertrophy (RVH). Hyperoxic-exposed rats had significant RVH as indicated by the increase in RV/LV+S ratio compared with room air control rats. H_2_S significantly reduced RVH (n = 6 animals per group, *P<0.005 hyperoxia vs. other groups). (**F**) Treatment with GYY4137 significantly attenuated PDGF-induced proliferation (n = 6/group, *p<0.001).

PHT is a significant complication in severe BPD. Neonatal rats exposed to chronic hyperoxia developed PHT as demonstrated by a significant decrease in the PAAT/RVET on echodoppler (Figure 4A, B) and an increase in MWT of small pulmonary arteries (Figure 4C, D) and RVH (Figure 4E). H_2_S attenuated these functional and structural features of PHT as indicated by an increase in mean PAAT/RVET (Figure 4B), a decrease in MWT (Figure 4D), and a reduction in RVH (Figure 4E). *In vitro,* ***t***reatment with GYY4137 significantly attenuated PDGF-induced PASMC proliferation (Figure 4F).

### GYY4137 Treatment Rescues the Loss of Alveoli after Established O2-induced Hypoalveolarization

**Figure 5:**
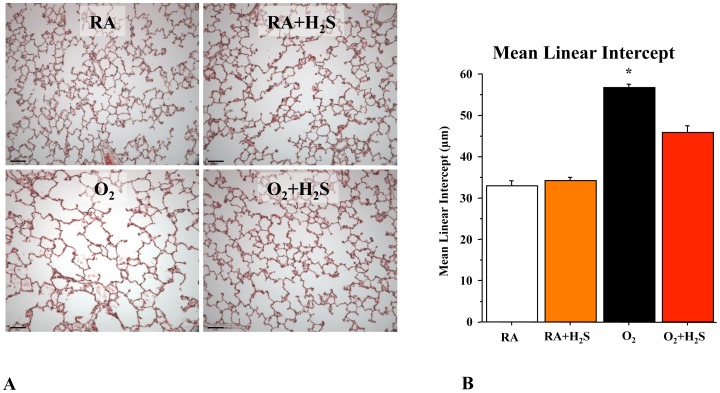
H_2_S rescues alveolarization after established O_2_-induced lung injury. (**A**) Representative H&E-stained lung sections of animals treated with GYY4137 from day P14–P24, after established lung injury, and harvested at P30. H_2_S in O_2_-exposed animals restored alveolar growth. (**B**) This is confirmed by the mean linear intercept (n = 5, *P<0.0001 hyperoxia vs. other groups, scale bar 65 µm).

We also tested the ability of GYY4137 to restore lung hypoalveolarization after established lung damage (Figure 5A). In this rescue experiment, untreated hyperoxia-exposed rats exhibited persistent impairment in alveolar growth (Figure 5A). GYY4137 administration from P14 (end of O_2_ exposure) to P24 significantly improved the lung architecture compared with O2-exposed animals (Figure 5B).

### GYY4137 Treatment Activates the PI3K Pathway and Decreases Apoptosis in O_2_-induced Lung Injury

**Figure 6:**
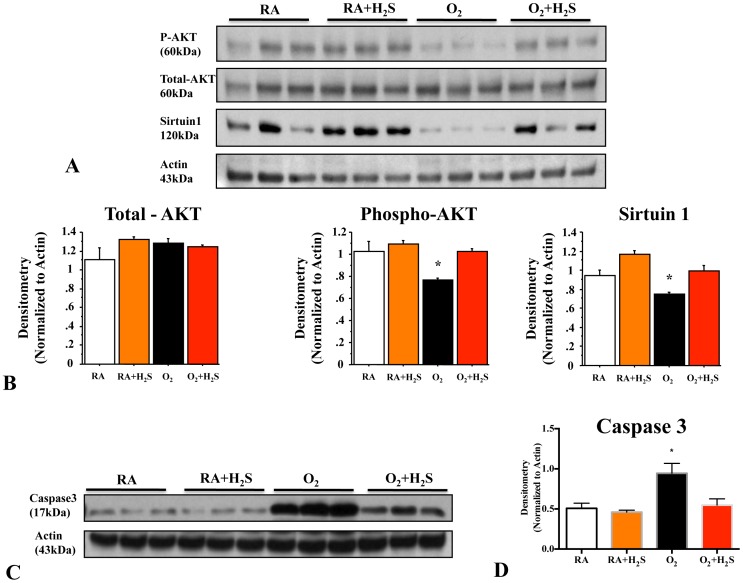
GYY4137 treatment activates the PI3K pathway and decreases apoptosis in O_2_-induced lung injury. (**A**) Immunoblots show decreased P-Akt and Sirtuin1 expression in hyperoxic-exposed lungs. (**B**) Treatment with H_2_S increased expression of P-Akt and Sirtuin1 expression in hyperoxic lungs (n = 3/group, *P<0.005). (**C**) *In vivo* H_2_S decreases apoptosis in oxygen-exposed lungs with BPD. Immunoblots of total caspase-3 and actin are shown for the four experimental groups. (**D**) Hyperoxia-exposed lungs showed increased total caspase-3 expression, which was attenuated by *in vivo* H_2_S treatment (n = 3/group, *P<0.005).

Hyperoxia significantly decreased whole lung P-Akt and Sirtuin1 expression (Figure 6A) and increased total caspase-3 (Figure 6C) expression compared with room air control lungs, suggesting decreased survival and enhanced apoptosis of lung cells. Conversely, treatment with GYY4137 preserved lung P-Akt and Sirtuin1 expression in hyperoxia (Figure 6B) and significantly attenuated apoptosis (Figure 6D).

### GYY4137 Preserves Mitochondrial Potential (ΔΨm) and Attenuates the Production of Mitochondrial Reactive Oxygen Species (mROS)

**Figure 7:**
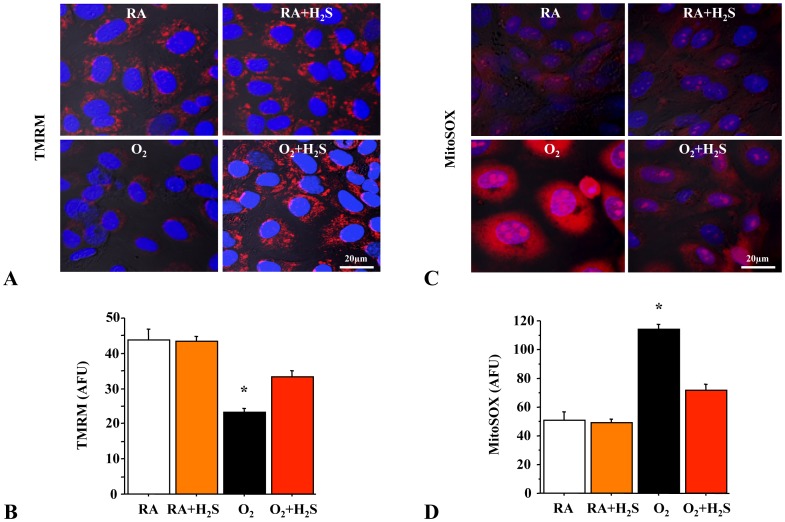
Rat lung epithelial cells (RLEs) exposed to hyperoxia decreased mitochondrial ΔΨm and increased mROS. Representative confocal microscopy at high magnification (×100) of rat lung epithelial cells (RLEs) showing (**A**) decreased ΔΨm (TMRM) and (**C**) increased mROS production (MitoSOX) in hyperoxia (TMRM and MitoSOX are in red, merged with nuclear stain DAPI in blue). Hyperoxia exposed RLEs treated with H_2_S have significantly increased ΔΨm (**B**) and decreased mROS (**D**) compared to hyperoxic control (n = 4 per group, *P<0.005 hyperoxia vs. other groups).

To further investigate the mechanism of GYY4137-induced lung cell survival, we assessed mitochondrial function in lung epithelial cells. Mitochondria are potential targets by oxygen radicals, and an alteration in mitochondrial membrane function is an important component of oxidative stress in cells. Because the mitochondrial membrane potential (ΔΨm) *in situ* is a measure of the energetic state of the cell as well as a sensitive indicator of mitochondrial function, we assessed the electrical potential across the inner mitochondrial membrane of room air and O_2_-exposed RLEs. RLEs exposed to hyperoxia had hypo-polarized mitochondria (decrease in ΔΨm) (Figure 7A, B) as assessed by tetramethylrhodamine methyl ester (TMRM) and increased mROS production (Figure 7C, D) as assessed by MitoSOX compared to room air controls. In contrast, treatment with GYY4137 attenuated the decrease in ΔΨ_m_ (Figure 7B) and dampened mROS production (Figure 7D).

## Discussion

Our findings demonstrate the protective effect of H_2_S on chronic hyperoxia-induced injury in the developing lung: *In vitro*, treatment with H_2_S protects HPAECs from O_2_ toxicity and promotes HPAECs network formation. *In vivo*, H_2_S administration preserves and restores alveolar growth and alleviates echographic and structural signs of PHT.

Similar to NO and CO, H_2_S is a small gaseous molecule generated in mammalian cells by enzymatic catalysis which diffuses freely across the membrane lipid bilayer [7]. In mammalian cells, H_2_S is produced from L-cysteine, catalyzed by one of two pyridoxal-5′-phosphate-dependent enzymes, cystathionine β-synthase (CBS) and/or cystathionine γ-lyase (CTH). H_2_S is considered a toxic gas. Its smell of rotten eggs can be perceived at concentrations as low as 0.0047 ppm. In serious cases, it causes cough, headache, pulmonary edema, or even coma. However, recent reports show that H_2_S is endogenously generated in the mammalian body and plays important physiological roles. Growing evidence implicates H_2_S in the pathogenesis of pulmonary diseases [17].

In the present study we show, both *in vitro* and *in vivo*, that H_2_S treatment displays lung-protective properties in the developing lung. Because angiogenesis contributes to alveolar growth [8], we examined the protective effect of H2S on HPAECs. *In vitro*, H_2_S preserved HPAECs viability and maintained HPAECs network formation in hyperoxia. Furthermore, H_2_S reduced HPAEC ROS levels in hyperoxia. This is consistent with reports showing that H_2_S protects cells and proteins from oxidative stress induced by peroxynitrite and hypochlorous acid [18]. In endothelial cells, hydrogen peroxide and organic hydroperoxides such as lipid hydroperoxides (LOOHs) are responsible for the activation of heme oxygenase-1 (HO-1), one of the ROS responders that trigger extensive oxidative damage in endothelial cells. H_2_S is capable of destroying hydrogen peroxide and LOOHs [19]. Consistent with these *in vitro* data, we show through vWF staining and CD31 lung protein expression that H_2_S preserved lung vascular growth in rats pups exposed to chronic hyperoxia.

Inhaled NO is a potent pulmonary vasodilator and promotes distal lung growth. Inhaled NO shows promise as a prophylactic therapy to decrease the incidence of BPD in experimental models [20]–[22], while results in preterm infants remain inconclusive [23]. Thus, we hypothesized that H_2_S would have similar beneficial effects on distal lung growth and PHT. *In vivo*, H_2_S indeed attenuated the arrested alveolar growth in the chronic oxygen induced arrested alveolar growth in rat model. While we demonstrate for the first time the protective effect of H2S on the developing lung, recent reports indicated a therapeutic potential of H_2_S in various acute adult lung injury models. Inhalation of 80 ppm H_2_S ameliorates lung pathology in LPS [24] and in ventilator [25] induced lung injury. Interestingly, Francis et al observed that 1 or 5 ppm H_2_S did not alter ventilation-induced lung injury, while 60 ppm H_2_S worsened ventilator-induced lung injury [26]. In contrast, intravenous pretreatment with sodium sulfide (Na_2_S) attenuated reduced pulmonary edema, enhanced the pulmonary expression of Nrf2-dependent antioxidant genes and prevented oxidative stress-induced depletion of glutathione in lung tissue [26]. This is consistent with the protective effect observed in the neonatal chronic hyperoxia-induced lung injury model, in which Nrf2 preserves alveolar growth while Nrf2 deficiency worsens lung injury [27], [28].

PHT often complicates chronic lung diseases including BPD and significantly worsens the prognosis [5], [6]. H_2_S induces vasodilatation and inhibits vascular smooth muscle cell proliferation [7]. In our study, hyperoxia-exposed rats exhibited marked PHT as assessed by echo Doppler (decreased PAAT/RVET), RVH, and remodeling of the pulmonary MWT. H_2_S alleviated these features of PHT, warranting further investigation of H_2_S as a potential treatment for PHT. The mechanisms by which H_2_S attenuates PHT, aside from increasing lung angiogenesis, remain unclear. To our knowledge, there are no data available on H_2_S interactions with the signaling pathways contributing to PHT in the hyperoxia model [29]. Interestingly, H_2_S protects against ballon injury induced neointima hyperplasia of the carotid artery and decreases vascular smooth muscle cell proliferation in this model [30]. Likewise, we found that GYY4137 attenuated PDGF-induced PASMC proliferation.

Interestingly, we showed that the expression of activated Akt *in vivo* decreased in the lungs of animals exposed to hyperoxia, while the expression of total caspase-3, a marker of apoptosis, significantly increased. Both these changes in P-Akt and total caspase-3 expression were significantly attenuated by H_2_S treatment. This observation indicates a potential role of the pro-survival PI3K/Akt pathway in determining the ability of AECs to resist to hyperoxic injury. H_2_S attenuates particulate matter–induced human lung endothelial barrier disruption via combined ROS scavenging and Akt activation [31]. Moreover, we have shown recently that activation of Akt protects alveoli from experimental oxygen-induced lung injury in newborn rats [9]. Accordingly, previous studies report that the H_2_S donor sodium hydrosulfide (NaSH) induces a dose and time-dependent increase in Akt phosphorylation in endothelial cells [32], which can be inhibited by the PI3K inhibitors LY 294002 and wortmannin. This suggests that H_2_S stimulates the activation of pro-survival Akt. Activation of Akt by various extracellular signals increases endothelial cell proliferation, migration, and tube formation *in vitro* [33], and mediates protective cytoskeletal rearrangement [34]. However, the mechanism by which H_2_S activates Akt is poorly understood and remains to be investigated.

We also found that sirtuin1 gene expression was higher in H_2_S-treated groups compared to untreated hyperoxia-exposed animals. Sirtuins (silencing information regulator) are nuclear nicotinamide adenine dinucleotide-dependent histone deacetylases. In mammalian cells, sirtuin1 appears to control the cellular response to stress by regulating the FOXO family of forkhead transcription factors. Because FOXO transcription factors transactivate a series of target genes that have critical roles in the cellular response to stress stimuli, endogenous sirtuin1 may potentiate FOXO’s ability to detoxify ROS and to repair damaged DNA [35]. It has been reported that sirtuin1 levels were reduced in macrophages and lungs of smokers and patients with chronic obstructive pulmonary disease due to its post-translational modifications by cigarette smoke-derived reactive components [36].

Lung cells exposed to hyperoxia can generate free radicals such as superoxide anion, hydroxyl, and alkyl radicals via mitochondrial electron transport [37]. Mitochondrial DNA, metabolism, and function are highly susceptible to ROS-induced injury. Such mitochondrial injury can contribute to the pathogenesis of necrotic and apoptotic cell death [38]. Whether enhancement of cell survival in this study is related to energy homeostasis and protection of mitochondrial function was investigated by measuring the ΔΨm of hyperoxia-exposed H_2_S treated and untreated cells. In our studies of ΔΨm, normoxia-exposed cells had higher arbitrary fluorescence units (AFU) values than hyperoxia-exposed cells. This could be due to hyperpolarization of the mitochondrial membrane caused by mitochondrial ATP accumulation in these metabolically inactive, growth-retarded cells, which may also have diminished ATP turnover. In addition, there was a remarkable increase in AFU observed in hyperoxia-exposed H_2_S-treated cells compared to untreated hyperoxia cells.

In conclusion, we show that H_2_S preserves and restores normal alveolar development and attenuates PHT in an experimental, oxygen-induced model of impaired alveolar development mimicking BPD. H_2_S may offer new therapeutic options for lung diseases characterized by alveolar damage and PHT and warrants further investigation.
